# Associations of maternal diet quality with weight gain during pregnancy and obesity at three-year postpartum in Jakarta

**DOI:** 10.1371/journal.pone.0244449

**Published:** 2020-12-31

**Authors:** Lestari Octavia, Rina Agustina, Arindah Nur Sartika, Annisa Dwi Utami, Yayang Aditia Dewi, Anastasia Hayuningtyas, Mutia Winanda, Erfi Prafiantini, Asmarinah

**Affiliations:** 1 Department of Nutrition, Faculty of Medicine, Universitas Indonesia–Dr. Cipto Mangunkusumo General Hospital, Jakarta, Indonesia; 2 Human Nutrition Research Center, Indonesian Medical Education and Research Institute (HNRC-IMERI), Faculty of Medicine, Universitas Indonesia, Jakarta, Indonesia; 3 Gunadarma University, Depok, Indonesia; 4 Department of Biology, Faculty of Medicine, Universitas Indonesia, Depok, Indonesia; Yenepoya Medical College, Yenepoya University, INDIA

## Abstract

Dietary changes during pregnancy (DP) and post-partum are essential for women’s nutrition status and the health of their offspring. We compared the diet quality DP and at 3-year post-partum (3YPP) and assessed the relationship between maternal diet quality and nutritional status using a prospective cohort design among women in East Jakarta. In total, 107 women were recruited from the study in 2014 and followed up at 3YPP in 2018. The mid-upper arm circumference (MUAC), weight, and height were evaluated. Food consumption data were collected from repeated 24-h recalls. The validated US Diet Quality Index-Pregnancy (DQI-P) scores with eight components were calculated using the food consumption data and compared between DP and 3YPP. Associations of the DQI-P score with the MUAC and weight gain DP and body mass index (BMI) at 3YPP were analyzed using multivariable linear and logistic regression. The median of the DQI-P score DP was significantly higher than at 3YPP [35 (27; 42) versus 27 (19; 30); *p*-value <0.001, respectively]. The higher DQI-P score was associated with increased weight gain DP of 3.3 kg (adjusted β = 3.30, 95% confidence interval = 1.06–5.54) after adjusting for the mother's age and household income. The DQI-P score was not associated with an increased risk of chronic energy deficiency DP and overweight–obesity at 3YPP. Thus, the diet adequacy was associated with weight gain DP but did not affect the MUAC DP and BMI at 3YPP. The DQI-P score DP was slightly better than the diet at 3YPP; however, the overall diet quality was inadequate. In conclusion, a higher DQI-P score was associated with increased weight gain DP of 3.3 kg but was not associated with other nutritional status indices in DP and 3YPP. Innovative dietary quality improvement programs are required to reduce malnutrition risk in pregnant and reproductive-age women.

## Introduction

Indonesia is among the low-middle-income countries (LMICs) suffering from a double burden of malnutrition. According to the Indonesian national health research conducted in 2018, the prevalence of child stunting is high, which is about 30.8% among under-5-year-old and 29.9% among below-2-year-old children [1]. In Jakarta, The prevalence of obesity among adults was 29.8%, which is higher than the national level of 21.8%. The prevalence of overweight–obesity in Jakarta increases from 34.8% in the year 2013 to 45.4% in the year 2018 [2]. The double burden of malnutrition increases the risk of having non-communicable diseases (NCDs) related to unhealthy and poor diet quality [3–5]. It indicates that lifestyle improvement is more likely to be developed since early life, conception, and pregnancy. However, longitudinal information on dietary changes from maternal to post-partum is not frequently reported, especially in LMICs.

Nutrient deficiencies during pregnancy (DP) are susceptible to the deterioration of the function and structure of the organ of the conceived fetus [6]. Obesity in the born child and later in his/her adult life is particularly associated with micronutrient, protein, and energy deficiencies throughout the pregnancy period [7]. Furthermore, short- and long-term detrimental health outcomes for the mother and her offspring are attributed to the excessive weight gain DP [8]. Therefore, inadequate maternal diet during the pre- and postnatal periods and its impact on nutritional status are crucial for reduction of the risk of global malnutrition.

In 2014, the Indonesian Ministry of Health developed practical guidelines for a healthy and balanced diet, as many other countries did. The establishment of guidelines helps in increasing awareness of the standard quality and quantity of nutrients for daily requirements and recommendations DP. In addition, these guidelines help pregnant mothers consume macro- and micronutrients sufficiently for an appropriate fetal development [9]. Sin et al. [10] reported that the diet quality and nutritional biomarkers during gestation were negatively associated with the weight status before pregnancy. It indicates that the period preceding pregnancy has been given less attention and is crucial to maximizing diet to reinforce fetal growth and development [11].

Indonesia has implemented various prenatal programs for supplementary feeding to reduce protein-energy malnutrition as well as supplementation and fortification programs to compensate for micronutrient shortages [12]. However, health problems concerning nutrition deficiencies remain. This situation indicates the importance of investigating the food nutrients and nutrient requirement recommendation for Indonesian women to provide data on consumption DP and years after post-partum. The longitudinal study to define the dietary quality DP and afterward will help design an effective program for the selected target group.

The study on diet quality among pregnant women is scarce, even more scarce is the study that compares the diet quality DP and at 3-year post-partum (3YPP). The use of the DQI-P as a tool to obtain the composite of the nutrients and food groups is appropriate as specific requirements for gestational diet is derived from the Pregnancy, Infection, and Nutrition (PIN) study [13]. The modified DQI-P also enables assessing the essential micronutrients required for different target groups [14]. Diet quality can also be utilized to determine the indices of nutritional status and biomarker during the gestation period [10].

The quality of diet and gestational weight gain contributed to maternal and newborn health outcomes [15]. The longitudinal method is a more practical ways to describe the change in diet quality DP and at 3YPP. Thus, we calculated the DQI-P score of mothers DP and at 3YPP and assessed its association with the nutritional status, especially with the weight gain DP, and the risk of having post-partum obesity in selected urban areas Jakarta, Indonesia.

## Materials and methods

### Study design and population

This study was part of the East Jakarta Cohort Study conducted by the Department of Nutrition, and Human Nutrition Research Center, IMERI, Faculty of Medicine, Universitas Indonesia. The first data collection was performed in 2014, which involved pregnant mothers in their third trimester of gestation, as described elsewhere [16, 17]; the follow-up collection was performed in 2018.

The inclusion criteria in the first recruitment were gestational age >32 weeks, apparently healthy pregnant women aged 19–40 years old, women who had planned to have delivery in Primary Health Centres (PHCs), and women who are willing to participate in the study. The exclusion criteria were mothers with chronic diseases, namely HIV/AIDS and tuberculosis, as seen from the medical record, and those who refused to participate in the study [17]. The gestational age DP was measured from the last menstrual period (LMP) to the delivery date, recorded in weeks. The gestational age was calculated from LMP from the health record, but the precise gestational age data in the first trimester was not recorded. Pregnant women in their third trimester were included in the study to ensure that the dietary pattern and mandatory supplementation from our government, such as iron-folic acid (IFA), was given to the target group. Furthermore, the dietary intake in the early trimester of pregnancy was varying due to vomit and nausea [18]. According to Nehring et al. (2011) [19], gestational weight gain was associated with post-partum weight retention in a prolonged post-partum period. A 3 to 5-year postnatal period is likely an appropriate time point for describing the weight gain retention after long periods of delivery. Post-partum duration of fewer than 3 years is insufficient to describe weight retention based on the US Institute of Medicine (IOM) pregnancy guidelines [19].

The subjects included in the 2018 data collection were those who had antenatal care in PHCs in the 2014 data collection within the living area, those who were residing in East Jakarta with low- to middle socioeconomic levels, and those who were willing to participate in the repeated data collection study. The 2014 initial sample consisted of 313 pregnant mothers; however, in the 2018 follow-up, only 40% (127) of the subjects were assessed.

**Fig 1:**
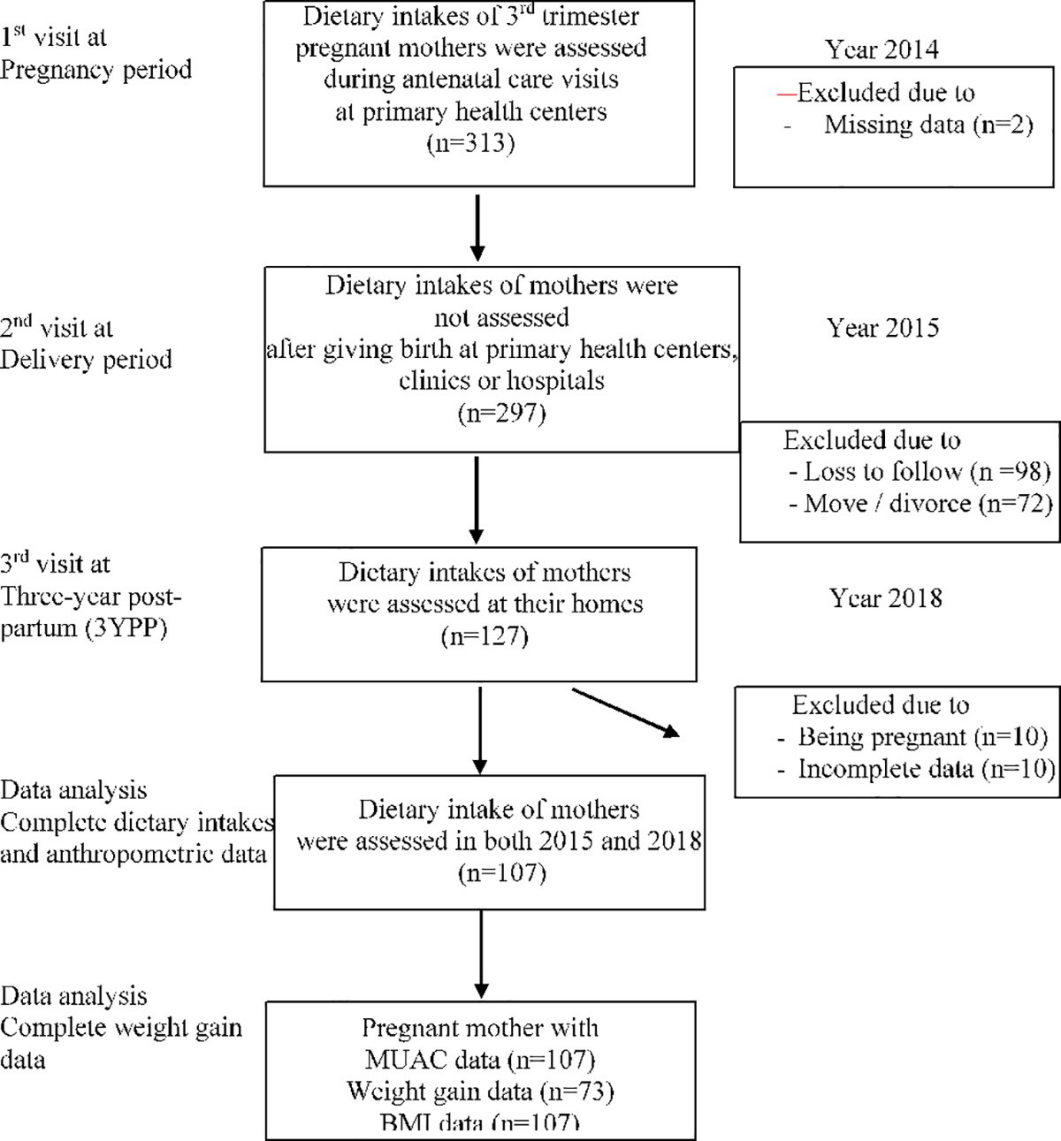
Subject recruitment and assessments of two times 24-h recalls in DP and 3YPP periods in urban areas of East Jakarta.

The migration rate of the participants to other cities or islands was 22.9%, whereas, for 36.6% of the subjects, their current addresses were not located. For the data analysis, ten mothers were excluded from the study due to subsequent pregnancy in 2018. Conclusively, the data of 214 and 107 subjects were available in 2014 and 2018, respectively. Data on weight gain DP were recorded only for 73 mothers due to failure in initial weight (first trimester) retracing (**Fig 1**). The 107 subjects analyzed from the 2015 and 2018 data, and they still represent the total subjects recruited in the study, as described in the result section, although loss to follow-up was 40%.

A total of 107 subjects have fulfilled the required of the minimum sample size (n = 90) for detecting the differences in weight gain DP between two dependent groups. The minimum sample size was calculated to compare the mean difference of the weight gain during two time periods using two-sided; (alpha = 0.05; 0.8 power, assumed 0.3 effect size), rounded up to calculate for a potential 20% dropout, according to Kac et al. **[36]**. We performed a post-hoc sample size calculation for a weight gain outcome, a total of 73 subjects have fulfilled the required minimum sample size with a power of 83% (alpha = 0.05, 0.35 effect size, two-sided).

### Data collection and socioeconomic variables

We used structured questionnaires to obtain information on general sociodemographic characteristics. Moreover, we collected data on the anthropometric measurements (body weight, body height, and mid-upper arm circumference (MUAC)). The questionnaire was also administered to collect data on dietary intake. The questionnaires, interviews, and assessments were pre-tested by trained enumerators one month before the commencement of the study. This activity involved 30 mothers living in the urban setting of Jakarta who had similar characteristics to primary study subjects but was not included in the main study. Pre-testing was conducted to evaluate the interviewer's ability to use the questionnaire and to define the effective ways to deliver the questions. It was also undertaken to assess the subject's response to the questions, especially for ambiguous terms, to harmonize technique and portion size of the dietary intake assessments, and to simulate filed data collection management. Furthermore, we calculated a Cronbach's alpha (0.78) to measure the questionnaire's reliability.

This study recruited trained enumerators to conduct the data collection in the field to collect socioeconomic variables that were defined as possible covariates. Such variables included family type, household income, schooling year attended, and mother's age. We categorized the family type based on nuclear and extended family. A nuclear family is defined as a family consisting of mother, father, and children, whereas extended family refers to those living with other members (uncle, niece, grandmother, etc.). Household income was divided based on the minimum provincial wages within those two time periods and converted to US dollar. The schooling year was classified based on the minimum period for education required by Indonesia, which is 9 years; less than nine years is considered as low education [20].

### Diet and nutritional status assessment

The repeated 24-h recalls, which consisted of one weekday and one weekend, were performed to collect data on the mothers’ food consumption. We identified 219 foods and 41 beverages consumed in both years, mostly from the processed food type. The validated food photographs captured by the Indonesian Ministry of Health were utilized to determine the standardized portion [21, 22]. If the food was not available on the list, a new recipe was developed; its ingredients, quantity, and processing method were inserted. The Nutrisurvey 2004 software was used to calculate the nutrient contents of the food consumed [23]. The software quantified the total energy and nutrient contents of the food; also, the calculation includes not only the processing method but also the contents of the raw ingredients.

The recommended serving size per day for Indonesians based on the available healthy and balanced diet guideline is three to four portions for grain, two to three portions for vegetables, and three to four portions for fruits. One portion of grain is equal to 100 g rice = 3/4 glass, one portion of fruit is 50 g = one slice of papaya, and one portion of vegetables is equal to 100 g = 1 glass of edible portion of vegetables [24]. In the absence of nutrient contents from the Indonesian food database, we utilized the nutrient content data from the US Department of Agriculture and Energy, and Nutrient Composition of Food, Health Promotion Board, Singapore, to estimate the nutrient contents. The portion size of the new recipe was converted into grams using Indonesia's food exchange list. The food groups and nutrients consumed by the respondents were also entered into the software. Subsequently, both nutrients from the recipes and the food groups were utilized to calculate the DQI-P score.

In minimizing the bias during the data collection of the dietary intake, we recruited and conducted the refreshing training for the enumerators with nutrition background. Moreover, we performed the pre-testing of the dietary intake questionnaire before data collection. We also assigned the same enumerator to re-collect the data obtained from the same subject, standardized the meal portion by using colored pictures of different portion sizes, and used the Indonesian Food Composition Table to calculate the nutrient content of the food consumed.

Subsequently, MUAC measurements were performed to evaluate the nutritional status of the mother DP using a standardized measuring tape, SECA 201, with 0.1 cm precision. The MUAC cut-off was set to <23.0 cm, which indicates chronic energy deficiency (CED) and ≥23.0, which means non-chronic energy deficiency (non-CED) [25]. The mother’s weight during the third trimester (within 32–40 weeks) and at 3YPP were measured using SECA^®^ digital 770; then, the result was rounded off to the nearest 0.1 kg. The height was measured using Shorrboard^®^, a portable height measuring board, and recorded the measurement to 0.1 cm. In height and weight measurement, the enumerator asked the subjects to remove the jacket, shoes, and possible light clothing wore. The body mass index (BMI) was calculated by dividing the weight (kg) by height (m^2^). Besides, BMI calculation was performed for the nutritional status of the mother pre-pregnancy and at 3YPP. Overweight and obesity were indicated by BMIs ≥25 and >30 kg/m^2^, respectively [26], whereas the weight gain DP was calculated by subtracting the weight during the first trimester from that during the third-trimester weight according to the IOM pregnancy guidelines [27]. The weight gain data during pregnancy were complete for 73 subjects, and the cut-off for this variable was based on the mean weight gain for pregnant women, as proposed by the IOM pregnancy guidelines. The categories of weight gain DP were divided based on the range of weight recommended by the IOM pregnancy guidelines. Those with BMI pre-pregnancy <18.5, 18.5–24.9, 25–25.9, and >30 kg/m^2^ are suggested to have gained weight DP of 12.7–18.1, 11.3–15.9, 6.8–11.3, and 4.9–9.1 kg, respectively [28]. We categorized the subject into insufficient, sufficient-excessive weight gain if the gaining of the weight below, within-above the range of recommended weight gain. The prevalence of obesity in 2014 was calculated from pre-pregnancy BMI and applied for 73 subjects.

### Diet Quality Index for Pregnancy (DQI-P)

The DQI-P tool was adopted from the PIN study in the USA [13], which consists of eight components, namely percentage (%) of the recommended serving of (1) grain, (2) vegetables, and (3) fruit; the percentage of the daily recommended dose for (4) folate, (5) iron and (6) calcium; the percentage of energy from (7) fat and (8) meal; and snack patterns score. Each component was converted to a DQI-P score, ranging from 0 to 10, with a maximum cumulative score of 80. A component is scored 0 if a certain type of food is not consumed and ten if the consumption meets the recommended serving, and the intermediate score is calculated proportionally [29]. In selecting this instrument, the need to emphasize on specified micronutrients, e.g., folate, should be considered, which is accommodated by this instrument’s ability to modify the calculations for the chosen nutrients [30]. Although the DQI-P is not a standard tool for measuring post-partum intake, this study aimed to compare the longitudinal diet quality for the same individual, thus allowing a similar instrument.

The consumption of grain, vegetables, and fruit transformed to score after calculating the recommended serving size in Indonesia's guidelines for a healthy and balanced diet. Dietary folate intake was expressed as a dietary folate equivalent (DFE). The source of nutrients were folic acid supplement, folic acid with food, and folate that is naturally found in food. The combination of these sources, 1.7 x folic acid (μg) + folate (μg) was noted as DFE [31, 32]. The folic acid and folate contents were obtained from the database or software calculation. Then, the levels of total folate, iron, and calcium were measured as percentage of the recommended dietary allowance (RDA) for age and pregnancy status. Based on Indonesia's healthy and balanced dietary guidelines, the RDAs for folate, iron, and calcium are 600 vs. 400 μg, 27 vs. 26 mg, and 130 vs. 110 mg for women in their third trimester pregnancy period and at reproductive age, respectively. Subsequently, the percentages of total folate, iron, and calcium were converted to scores. In DQI-P, the total fat was defined as the amount of fat consumed in total energy intake, whereas ≤30% of the energy intake coming from fat was categorized as a maximum score. The highest score of the meal pattern was identified as three times of meals and two times of snacks. The DQI-P distribution was usually displayed as the mean value of the component. The original study divided the scores into eight categories due to the equal distribution of the scores above the mean score [13]. In our study, we divided the DQI-P scores based on 40% of the total score into two groups (score of ≤32 and >32) for both years of observation. This approach was taken as most of the score was below the median/mean of the total score. This presumption number is appropriate for Indonesian women rather than the median of the total score and is adjusted to equally distribute the score within the population [18]. Furthermore, the cut-off score (i.e., 32) was used to equally distribute the number of subjects in two groups because most of the subjects had lower total score (<40) of total DQI-P. The detailed components of DQI-P, the number of subjects, and the median of each category were presented in the result section.

### Statistical analysis

Statistical Package for Social Science version 20 was used for data analysis. The normal distribution of the data was performed using the Kolmogorov–Smirnov test. Normally distributed data were expressed as mean ± standard deviation (SD), whereas the median and interquartile ranges were applied for non-normally distributed data. Descriptive analysis was conducted for all variables to obtain data from the profiles of the subjects. In contrast, the Mann–Whitney U test was employed to compare the difference for non-normally distributed data. Initially, the possible covariates were calculated using bivariable logistic regression to determine the unadjusted odds ratio (OR) and 95% confidence intervals (CI) of each outcome (non-CED, sufficient–excessive weight gain DP, and overweight–obese at 3YPP).

The main predictor of DQI-P was categorized into two groups. The other predictors that were potential to be confounders, were determined in the analysis, that had the *p*-value of <0.25 based on bivariate analysis [33]. *P*-value <0.05 was applied to determine the statistical significance, and 95% CI did not include 1. In the multivariable logistic regression, all the potential covariates (i.e., age and household income) were included in the multivariate analyses to account for the effect of covariates on the model. Logistic regressions were conducted to determine the possibility of the DQI-P score to affect not being CED, not having sufficient–excessive weight gain DP, and not being overweight–obese at 3YPP.

The overweight and obesity classifications based on BMI calculation were categorized in one group for the association between diet quality and obesity. Thus, linear regression was conducted to measure the possibility of the associations of the DQI-P score with MUAC, weight gain during DP, and BMI. In the linear regression analysis, age was included as a covariate that affected the dependent variable.

### Ethical clearance

As previously described by Angkasa and colleagues [17], the approval of the study in 2014 was obtained from the ethics committee of the Faculty of Medicine Universitas Indonesia and Dr. Cipto Mangunkusumo General Hospital, Jakarta, Indonesia, with the serial number 859/UN2.F1/ETIK/2014. In addition, the follow-up study conducted in 2018 obtained the ethical clearance from the same committee, with the serial number 0420/UN2.F1/ETIK/2018. Both studies were permitted by the local authority of East Jakarta District, District Health Office of East Jakarta, and the PHCs in subdistricts. The registration number of the research protocol to clinicaltrials.gov is NCT04096521. The current study included mothers in the previous study. We provided written information and made the research team available for further information required. The approval for study participation was obtained by signing the given form.

## Results

### Characteristics of the subject

The socioeconomic characteristics of the subject are presented in **Table 1**. The mean age of the subjects was 29.1 and 33.1 years old in 2014 and 2018, respectively. Most of the mothers accomplished their 9 years of schooling. The percentage of families who live below the provincial minimum wage was higher in 2018 compared with 2014.

**Table 1:** Characteristics of the women recruited in the initial study and subjects included during pregnancy (DP) and at 3-year post-partum (3YPP) in the selected urban areas of East Jakarta.

Variables	Period		
	Total number of subjects (2014)	DP (2014)	3YPP (2018)
	(n = 313)	(n = 107)	(n = 107)
**Age (years)**^**1**^	28.7 ± 5.37	29.1 ± 5.44	33.1 ± 5.24
**Family type, n (%)**			
** Nuclear**	186 (59.4)	60 (56.1)	63 (58.9)
** Extended**	127 (40.6)	47 (43.9)	44 (41.1)
**Median household income (USD)**^**2**^^,^^**3**^	186 (143; 250)	179 (143; 243)	245 (210; 350)
**Level of household income, n (%)**^**4**^			
** ≤ low**	172 (55.0)	61 (57.0)	60 (56.1)
** ˃ sufficient**	141 (45.0)	46 (43.0)	47 (43.9)
**Schooling year attended, n (%)**			
** <9**	95 (30.4)	8 (7.1)	8 (7.1)
** ≥9**	218 (69.6)	99 (92.9)	99 (92.9)
**CED, n (%)**	20 (6.3)	8 (7.5)	5 (4.7)
**Overweight and obesity, n (%)**^**Ƨ**^	78 (34.2)^¥^	59 (80.8)^5^^,ω^	69 (64.5)

Note: DP indicates during pregnancy, and 3YPP indicates 3-year post-partum.

^1^Values are expressed in mean ± SD.

^2^Values are expressed in median (25^th^;75^th^ percentile).

^3^1 USD = Rp 14,000 in 2014 and 1 USD = Rp 14,300 in 2018.

^4^Provincial minimum wages in 2015, Rp 2,700,000 = USD 192.86; provincial minimum wages in 2018, Rp 3,648,035 = USD 255.

^Ƨ^Cut-off for overweight and obesity indicated by a BMI ≥25 kg/m^2^ based on the pre-pregnancy calculation.

^5^Data available for 73; the median gestational age at the third trimester was 35 (34; 36)^2^ weeks, where as the gestational age at the first trimester was within 6–12 weeks.

^¥^Data available for 228.

Moreover, the prevalence of CED was low for both years, 2014 and 2018, whereas most overweight and obesity reached 80.8% for the pre-pregnancy BMI and was 64.5% at 3YPP. In 2014, the prevalence of obesity was calculated from the pre-pregnancy BMI for 73 subjects. Those included in the analysis share the same characteristics with the subject who were excluded in the assessment.

### Predicted risk factors related to the outcome observed

**Table 2** presents the possible risk factors related to non-CED, sufficient, and excessive weight gain DP and BMI at 3YPP. Of the 73 subjects who recorded their weight measurements, the household income was indicated to contribute to insufficient and excessive weight gain DP (*p*-value <0.05) and was considered as a confounder. In contrast, the other variables did not indicate the possibility of observed outcomes.

**Table 2:** Sociodemographic and economic risk factors for non-chronic energy deficiency (CED) DP, weight gain DP and not overweight-obese at 3YPP.

Risk factors	Non-CED DP (n = 107)^6^			Sufficient and excessive weight gain DP (n = 73)^7^			Not overweight-obese at YPP (n = 107)^8^		
	n (%)	Unadjusted OR (95%CI)	*p-*value	n (%)	Unadjusted OR (95%CI)	*p*-value	n (%)	Unadjusted OR (95%CI)	*p*-value
**Family Type**									
** Extended**	44 (44.4)	1.00		14 (46.7)	1.00		24 (63.2)	1.00	
** Nuclear**	55 (55.6)	1.33 (0.30–5.89)	0.76	16 (53.3)	0.99 (0.39–2.53)	0.99	14 (36.8)	0.94 (0.42–2.10)	0.90
**Household income**									
** ≤low**	55 (55.6)	1.00		12 (40)	1.00		24 (63.2)	1.00	
** >low**	44 (44.4)	2.40 (0.46–12.48)	0.30	18 (60)	3.46 (1.3–9.2)*	<0.05	14 (36.8)	0.64 (0.28–1.43)	0.28
**Schooling year attended**									
** <9**	6 (6.1)	1.00		1 (3.3)	1.00		0 (0)	1.00	
** ≥9**	93 (93.9)	0.95 (0.22–4.22)	0.95	29 (96.7)	0.69 (0.04–11.49)	0.80	38 (100)	7.88 (0.43–143.84)^9^	0.16
**Mother's age in years**									
** ≤31**	63 (63.6)	1.00		22 (73.3)	1.00		18 (48.6)	1.00	
** >31**	36 (36.4)	0.95 (0.21–4.22)	0.95	8 (23.7)	0.56 (0.20–1.53)	0.26	19 (51.4)	0.53 (0.23–1.19)	0.13

CED, chronic-energy deficiency; DP, during pregnancy; 3YPP, 3-year post-partum.

^6^reference: CED.

^7^reference: not sufficient weight gain.

^8^reference: overweight-obese.

^9^run with firth logic using STATA.

* significant *p*-value <0.05, logistic regression test.

*p*-value < 0.25, possible confounders in the study.

### Dietary component included in the DQI-P

**Table 3** presents the variables calculated in the DQI-P. Three out of eight variables can meet the maximum score, namely grain, meal pattern, and percentage of body fat to total energy intake. Those variables are most likely to reach the maximum score (10), yet the highest scores of the DQI-P is obtained by meal pattern calculation for both 2014 and 2018. The percentage of the subjects who consumed the maximum score of grain is more than 40%. Moreover, the snack and meal score is even higher, which is up to 56%. The maximum score for vegetables, fruits, iron, folate, iron intake, and calcium DP and at 3YPP is achieved by 1.9% vs 2.8%, 12.1% vs 0%, 0.9% vs 9.3%, 1.9% vs 3.7%, and 50.5% vs 0%, respectively.

**Table 3:** Dietary components included in the diet quality index for pregnancy (DQI-P) DP and at 3YPP.

Dietary component	Score	Score categories	DP period	3YPP period
		(% recommended portion)	2014	2018
			n (%)	n (%)
**Grains**	10	≥ 100	59 (55.1)	44 (41.1)
	5	50–99	46 (43.0)	52 (48.6)
	0	< 50	2 (1.9)	11 (10.3)
**Vegetables**^**10**^	10	≥ 100	2 (1.9)	3 (2.8)
	5	50–99	14 (13.1)	20 (18.7)
	0	< 50	91 (85.0)	84 (78.5)
**Fruits**^**10**^	10	≥ 100	13 (12.1)	0 (0)
	5	50–99	19 (17.8)	2 (1.9)
	0	< 50	75 (70.1)	105 (98.1)
**Folate intake**^**10**^	10	≥ 100	1 (0.9)	10 (9.3)
	5	50–99	44 (41.1)	32 (30.0)
	0	< 50	62 (58.0)	65 (60.7)
**Iron intake**^**10**^	10	≥ 100	2 (1.9)	4 (3.7)
	5	50–99	5 (4.7)	5 (4.7)
	0	< 50	100 (93.4)	98 (91.6)
**Calcium intake**^**10**^	10	≥ 100	54 (50.5)	0 (0)
	5	50–99	36 (33.6)	4 (3.7)
	0	< 50	17 (15.9)	103 (96.3)
**% of fat to total energy**	10	≤ 30	42 (39.3)	43 (40.2)
	7	30–35	36 (33.6)	30 (28)
	4	35–40%	14 (13.1)	17 (15.9)
	0	>40	15 (14.0)	17 (15.9)
**Meal pattern, meal/snack occasion**	10	3 / 2	77 (72.0)	56 (52.4)
	5	3 / 0–1 or	27 (25.2)	44 (41.1)
		2 / 2		
	0	2 / 0–1 or	3 (2.8)	7 (6.5)
		1 / 1		

DP, during pregnancy; 3YPP, 3-year post-partum.

^10^score average from dietary intake data.

### DQI-P calculation for both 2014 and 2018

**Table 4** presents the data in the DQI-P calculation for both pregnancies (2014) and post-partum (2018) periods. The median (25^th^; 75th percentile) of the DQI-P scores in 2014, 2018, and overall periods was 35 (27; 42), 27 (19; 30), and 40 (20; 60), respectively. Subsequently, the subjects were divided based on 40% of the total score into ≤32 and >32. A 40% cut-off was used to enable the equal distribution of the group. During the pregnancy period, 62 out of 117 mothers had a score >32. At 3YPP, the score was lower than the score DP by more than 50% of the mothers.

**Table 4:** Median values of diet quality index (DQI-P) of the women DP (2014) and 3YPP period (2018).

Component	Total DQI-P score of the mothers								
	DP in 2014 (n = 107)			3YPP in 2018 (n = 107)			Two periods		
	Median (25^th^;75^th^ percentile)	(%)	(%)	Median (25^th^; 75^th^ percentile)	(%)	(%)	Median total	Median changes	*p-*values changes
	35 (27; 42)	≤ 32	>32	27 (19; 30)	≤32	>32	30	-8 (8; 12)	<0.001^§^
		(n = 45)	(n = 62)		(n = 86)	(n = 21)	(n = 214)		
**% serving per day**									
**Grain**^**11**^^,^ ^**18**^	100 (83.3;133.3)	83.3	116.7	100.7 (73.6;126.9)	90.2	124.0	100.2	+0.7	>0.05
**Vegetables**^**12**^^,^ ^**19**^	25.0 (25.0; 50.0)	25.0	25.0	22.7 (13.3; 40.0)	20.8	60.0	25.0	-2.3	>0.05
**Fruits**^**13**^^,^ ^**20**^	25.0 (0.0; 50.0)	25.0	25.0	1.4 (0;15.8)	1.2	8.0	15.0	-23.6	<0.001**
**% RDA**									
**Folate**^**14**^^,^ ^**21**^	100.9 (65.1; 135.0)	71.1	110.0	43.4 (29.1; 66.6)	39.3	74.9	66.3	-57.5	<0.001**
**Iron**^**15**^^,^ ^**22**^	25.5 (20.4; 29.9)	23.3	27.5	24.8 (16.6; 34.9)	22.0	34.9	25.3	-0.7	˃0.05
**Calcium**^**16**^^,^ ^**23**^	44.2 (27.6; 67.7)	30.5	55.1	17.7 (11.1; 24.4)	15.1	23.7	25.2	-26.5	<0.001**
**% of total energy from fat**	31.3 (27.0; 35.9)	31.7	30.5	31.5 (25.6; 38.4)	31.9	28.6	31.4	-0.2	˃0.05
Score meal pattern*^17^	10.0 (5.0; 10.0)	10.0	10.0	10.0 (5.0;10.0)	5.0	10.0	10.0	0.0	<0.05*

DQI-P, dietary quality index-pregnancy; DP: during pregnancy 3YPP, 3-year post-partum.

^11^6 servings grain per day, Indonesia guideline for a balanced diet for pregnant women.

^12^4 servings vegetables per day, Indonesia guideline for a balanced diet for pregnant women.

^13^4 servings fruit per day, Indonesia guideline for a balanced diet for pregnant women.

^14^Based on Indonesia's RDA of folate for pregnant women, 600 μg/day.

^15^Based on Indonesia's RDA of iron for pregnant women, 27 mg/day.

^16^Based on Indonesia's RDA of calcium for pregnant women, 1200 mg/day.

^17^3 times meals and two times snacks.

^18^4.5 servings grain per day, Indonesia guideline for a balanced diet for non-pregnant women.

^19^3 servings vegetables per day, Indonesia guideline for a balanced diet for non-pregnant women.

^20^5 servings fruit per day, Indonesia guideline for a balanced diet for non-pregnant women.

^21^ Based on Indonesia's RDA of folate for non-pregnant women, 400 μg/day.

^22^ Based on Indonesia's RDA of iron for non-pregnant women, 26 mg/day.

^23^ Based on Indonesia's RDA of calcium for non-pregnant women, 1000 mg/day.

* significant *p*-value <0.05, Mann–Whitney U test.

** significant *p*-value <0.01, Mann–Whitney U test.

### Logistic and linear regression of the DQI-P category and the outcome

**Table 5** demonstrates that mothers with a DQI-P score were not associated with being non-CED [AOR = 1.46, 95% CI (0.43–6.25)] and having sufficient-excessive weight gain DP [AOR = 1.01, 95% CI (0.39–2.65)]. Moreover, DQI-P also did not exhibit an association with the mothers not being overweight-obesity of the mothers at 3YPP [0.77, 95% CI (0.26–2.25)], even after being adjusted for age and household income for all the associations tested. **Table 6** demonstrates that the DQI-P score positively correlates with the weight gain DP [adjusted β = 3.30, 95% CI (1.06–5.54)]. The higher DQI-P score was associated with an increase in 3.3 kg of weight gain DP but not with other nutritional status indices in those two periods. However, DQI-P was not associated with MUAC, and the risk of being overweight and obese at 3YPP [adjusted β = -0.62, 95% CI = (-1.68–0.45), adjusted β = -0.32, 95% CI (-2.52–1.89)], respectively.

**Table 5:** Associations of diet quality score classification with a proportion of non-CED DP, non-low weight gain DP, and overweight and obese at 3YPP.

Variables	DQI-P score	n (%)	Yes (%)	UOR (95% CI)	*p-*value	AOR (95% CI)	*p-*value
**Non-CED DP (n = 107)**^**24**^	≤32	45 (42.1)	41 (41.4)	1.00		1.00	
	>32	62 (57.9)	58 (58.6)	1.42 (0.33–46)	0.64	1.46 (0.43–6.25)	0.61
**Sufficient–excessive weight gain DP (n = 73)**^**25**^	≤32	29 (39.7)	12 (40)	1.00		1.00	
	>32	44 (60.3)	18 (60)	0.98 (0.38–2.54)	0.98	1.01 (0.39–2.65)	0.98
**Not overweight–obese at 3YPP (n = 107)**^**26**^	≤32	86 (80.4)	31 (81.6)	1.00		1.00	
	>32	21 (19.6)	7 (18.4)	0.89 (0.32–2.43)	0.82	0.77 (0.26–2.25)	0.63

CED, chronic energy deficiency; DP, during pregnancy; 3YPP, 3-year post-partum.

DQI-P was set to 32 as 40% of the total score; UOR, unadjusted odds ratio; AOR, adjusted odds ratio.

^24^CED is determined by mid-upper arm circumference (MUAC) of < 23 cm.

^25^Based on the NC Department of Health and Human Services; Women's and 'Children's Health Section. Prenatal weight gain chart. Adapted from the Institute of Medicine, 2009. Weight gain during pregnancy: Reexamining the guidelines. Washington, DC. National Academies Press: Committee to Reexamine IOM Pregnancy Guidelines.

^26^Based on the body mass index for overweight and obesity ≥25 kg/m^2^.

**Table 6:** Associations of diet quality and mid-upper arm circumference and weight gain DP and body mass index during at 3YPP.

Variables	DQI-P score	n (%)	Unadjusted β (95% CI)	*p-*value	Adjusted β (95% CI)	*p-*value
**MUAC DP (n = 214)**	≤32	131 (61.2)	-0.75 (-1.81–0.30)	0.16	-0.62 (-1.68–0.45)	0.25
	>32	83 (38.8)	1.00		1.00	
**Weight gain DP**^**27**^ **(n = 73)**	≤32	29 (39.3)	2.85 (0.51–5.20)	0.02*	3.30 (1.06–5.54)	0.004**
	>32	44 (60.3)	1.00		1.00	
**BMI 3YPP (n = 107)**	≤32	86 (80.4)	-0.07 (-2.25–2.18)	0.95	-0.32 (-2.52–1.89)	0.78
	>32	21 (19.6)	1.00		1.00	

DQI-P, diet quality index; MUAC, mid-upper-arm circumference; BMI, body mass index; DP, during pregnancy; 3YPP, 3-year post-partum.

^27^the complete data set for weight gain was 73 subjects.

Adjusted by age and household income.

* significant *p-*value <0.05, linear regression analysis.

** significant *p*-value <0.01, linear regression analysis.

## Discussion

The current study found that a higher DQI-P score was associated with increased weight gain during pregnancy of 3.3 kg but was not associated with other nutritional status indices during pregnancy and at 3-year postpartum. The overall diet quality of women during pregnancy and at 3-year postpartum based on the DQI-P score calculation was insufficient to meet the recommended dietary requirement. The grain (most were white rice and flour-based food) and meal patterns were two variables that encountered the maximum score of 10 in the DQI-P calculation for the two periods, indicating adequate serving consumption of grain and frequency of meals and snacks. Diet quality during pregnancy was not associated with sufficient–excessive weight gain during pregnancy, either before or after adjustment by the age of the mothers and household income; moreover, it did not affect MUAC during pregnancy and BMI at 3-year postpartum. Although the prevalence of overweight and obesity was slightly higher at 3-year postpartum than pre-pregnancy, this study did not find a significant association between CED DP or being overweight–obese at 3-year postpartum. The higher DQI-P score will increase the weight by 3.3 kg during pregnancy after adjusting for the mother's age and household income. Indonesia's healthy and balanced diet guidelines have provided a practical recommendation for daily intake; however, the consumption was low either during pregnancy or at 3-year postpartum.

The PIN study developed the DQI-P score calculation [13] and yielded results that corroborate the findings of the previous works concerning the dietary assessment. In our study, the median score was 24 points lower from the mean score of the PIN study (32 vs. 56) [13], and 18.01 points lower than the total mean score of the study in Texas [29]. Within those two periods, the fruit, folate, and calcium intake were significantly higher DP than 3YPP; however, those components still did not meet the RDA [24]. The low score of DQI-P of the women in this study may be associated with the low affordability that restricts access to purchase qualified foods from the local market in their neighborhoods [34]. Besides, the DQI-P tool adopted in this study may not all be in line with dietary guidelines and the differences in the variety of food consumed in Indonesia. In applying DQI-P in the calculation of the diet quality in the USA, dietary intake and the nutrient requirement DP were used, as established by the United States Department of Agriculture [29]. Meanwhile, in this study, the calculation was based on adherence to the Indonesian balanced and healthy diet [24]. Stefani et al. summarized Indonesia and USA dietary guidelines presented as Healthy Eating Index which is closely similar in food groups and nutrient recommendation (Stefani et al., 2018, Dietary quality of predominantly traditional diets is associated with blood glucose profiles, but not with total fecal Bifidobacterium in Indonesian women, Plos One).

A Norwegian mother and child cohort study revealed that low adherence to healthy and balanced diet guidelines and recommended nutrient would increase the risk of excess weight gain, which tends to remain after pregnancy [35]. Contrarily, the findings in our study indicated that the diet quality DP did not exhibit any association between overweight**–**obesity at 3YPP. The inability to associate with overweight**–**obesity at 3YPP may be related to the scope of DQI-P, which fails to identify some nutrients that may contribute to positive energy balance such as red meat and sugar. The Norwegian study indicated that red meat, sugar, and sodium should be consumed in a limited amount [35]. A study on Brazilian women revealed that the 9-months post-partum weight retention was determined by several factors, namely socioeconomic, race, age at first delivery, gestational weight gain, and percentage of body fat. The Brazilian study revealed that a body fat percentage ≥30% at baseline would increase the risk of 7.5 kg or 10.2 times higher compared to those who had less body fat [36].

The highest score achieved within this study population was from meal grain consumed and eating frequency. Conversely, insufficient intake of fruits, vegetables, and micronutrients was observed within two periods. The women in this study consumed fruits, vegetables, and iron less than 30% of the recommended amount. A possible explanation for the slightly better diet quality DP compared with that at 3YPP is the impact from the existing program of the government, family support, and special attention given DP, which resulted in better compliance with Indonesia's dietary guidelines DP. Six serving portions of grain and four portions for vegetables and fruits for pregnant women are recommended to meet the macro- and micronutrient requirements for fetal development [9]. Furthermore, as a vulnerable group, pregnant women receive supplementation programs to prevent birth defects [37]. Consumption during gestation tended to be quite excessive, particularly for grain that accounted for more than half of the total consumption (53%), above the recommended serving portion. In Indonesia, the staple food consumed are refined grains, such as rice and flour; however, the intake of animal products, fruits, and vegetables is also low [38]. Inadequate intake of animal products could explain the low micronutrient content; contrarily, the high intake of processed and flour-based foods increased the oil, sugar, and fat that were low-nutrient energy-dense [38–40].

To our knowledge, our study is among the limited studies examining the diet quality of women between the DP and 3YPP periods in the low socioeconomic group of developing countries. The subjects recruited in this study are representatives of the population in the low and middle socioeconomic levels in the urban setting of LMICs [30]. This DQI-P tool was employed to measure the dietary quality of urban Indonesian women in a composite of macro- and micronutrient, not in a single nutrient source foods. The scoring system may not be sensitive enough to detect the relationships of all the measured nutritional outcomes. Still, it could provide data on the diet quality of women in a low-income society [41]. The limitation of this study is the lack of data on the diet quality of mothers during lactating periods. Moreover, another drawback of the DQI-P tool is the inability to determine the type of grain consumed, which underestimates the calculation [42]. To ensure the internal validity and quality of the study, the research team employed a standardized method consisting of well-trained enumerators, structured questionnaires, and food visualization tools to describe the amount consumed by the participants [22]. The validity of this tool is also adequate since it can detect the differences in the variables measured in two periods [43].

Overall, poor diet quality DP may increase the risk of weight gain DP. Obesity-related DP may be related to adverse birth outcomes [44] as well as the weight retention, which could lead to post-partum obesity [45]. A study conducted in Rotterdam, Netherland, revealed that the increase in weight gain DP is related to socioeconomic factors, such as minimum family income and low educational level, multiparous, and genetic factors [46]. The IOM pregnancy guidelines recommend that weight gain DP lies in the range of 11.3–15.9 kg, whereas for obese pregnant mothers, it ranges from 4.9 to 9.1 kg [28].

Poor diet quality is consistent in almost all components, except for the total fat that makes up ≤30% of the total energy and meal patterns. This data emphasizes the fact that serious problems existed in the adult dietary pattern, which needs to be solved with comprehensive actions. The pre-pregnancy and gestational periods need to be highlighted as modifiable factors for the mother and the children for long-term effects.

Further study should include more subjects with diversed socioeconomic levels and geographical areas from urban-rural settings. Enhancement in the socioeconomic status and eating behavior of the women at reproductive age would lead to better food intake and quality of life as well as reduce risk of weight retention after pregnancy in the future.

## Conclusions

A higher DQI-P score was associated with increased weight gain during pregnancy of 3.3 kg but was not associated with other nutritional status indices in during pregnancy and 3-year postpartum. Although the overall dietary quality of women was impoverished based on the DQI-P score during pregnancy and three-year post-partum, the diet quality during pregnancy was slightly better with that at three-year post-partum. The adequacy of DQI-P components contributed to a higher score of the diet quality index in terms of grain consumption food-based and frequency of meals and snacks and was associated with weight gain DP. The DQI-P score was not related to the risk of being overweight and obese at 3YPP. Further research and actions should be conducted and taken, respectively, to spread awareness of the meal pattern that helps combat the high prevalence of overweight and obesity, which would lead to a lower incidence of NCDs in Indonesia.

## Supporting information

S1 Data (XLS)
